# Narrative Tensions in Strained Junior Elite Performers’ Experiences of Becoming Elite Performers

**DOI:** 10.3389/fpsyg.2021.645098

**Published:** 2021-06-03

**Authors:** Heidi M. Haraldsen, Frank E. Abrahamsen, Bård Erlend Solstad, Hallgeir Halvari

**Affiliations:** ^1^Norwegian Research Center for Children and Youth Sports, Oslo, Norway; ^2^Department of Dance, Oslo National Academy of the Arts, Oslo, Norway; ^3^Department of Sport and Social Sciences, Norwegian School of Sport Sciences, Oslo, Norway; ^4^Department of Sport Science and Physical Education, University of Agder, Kristiansand, Norway; ^5^Department of Business, Marketing, and Law, University of Southeastern Norway, Hønefoss, Norway

**Keywords:** narrative theory, talent development, elite performance, identity, physical and mental health

## Abstract

Contextualized within narrative theory and the field of talent identification and development systems (TIDS), this interview study examined strained junior elite performers’ experiences of becoming elite performers while participating in prestigious national TIDS. The study explored how junior elite performers perceive and negotiate their personal narratives of becoming within a cultural master narrative of being. The focus is on how the quality of person-environment interaction, characterized by narrative alignment or tensions, relates to perceptions of identity, agency, and physical and mental health. We purposefully recruited eight participants (*M*age = 17.31, *SD* = 0.9) from a previously published study, who reported experiencing suboptimal psychological functioning compared with their peers to explore narrative tensions in their storylines. The data were collected through semi-structured interviews and examined, using narrative analysis. We identified “the performance narrative” as the dominating cultural narrative within the TIDS and three distinct personal narratives of negotiation with unique characteristics: obsessive and externally driven alignment – “striving to stay at the top of the game”; tensions – “just hanging in there”; and disruption from alignment – “when the going gets tough.” The results indicated that tensions and lack of alignment between the dominating cultural narrative and the individual narrative seem to increase the risk of experiencing identity challenges, suboptimal functioning, and aspects of ill-being. The study offers critical reflections on the dominating performance narrative within TIDS and additionally suggests an alternative athlete-centered and more holistic approach that combines both personal and performance developments.

## Introduction

Talent identification and development systems (TIDS) have, for centuries, been concerned with how to optimize performance potential and balance risks with rewards (Miller and Kerr, 2002; Côté and Gilbert, 2009). Research has shown that the performance-oriented nature of TIDS (i.e., early specialization, high expectations, and social isolation) is often in conflict with and comes at the expense of personal development and well-being (Barker-Ruchti and Schubring, 2016; Preston and Fraser-Thomas, 2018). Hence, nuanced evidence of how TIDS might build facilitative, in contrast to dysfunctional psychosocial learning climates, is required to foster more balanced and healthier TIDS (Rongen et al., 2014; Rice et al., 2016).

The process of becoming an elite performer is proposed to be situated and complex, with unique individual, situational, and contextual elements in play (Aggerholm, 2015; Stabell, 2018). Children and adolescents are not just passively socialized; instead, they display active attempts at the agency and social negotiation (Hansen and Jessop, 2017; Stabell, 2018; Ronkainen and Ryba, 2020). While some performers struggle, others seem to cope and thrive when faced with the high demands required to perform at the highest level (Houltberg et al., 2018; Stabell, 2018). Narrative research has indicated that a multifaceted identity, in contrast to a strong and exclusive performance identity, exhibits greater mental health, wellbeing, and resilience (Carless and Douglas, 2013b; Willard and Lavallee, 2016). However, the processes of how junior elite performers conceptualize and negotiate their situations differently are still underexplored (Ronkainen and Ryba, 2020; Scholtz, 2020). Additionally, a predominant focus has been on narratives of the past and of the perspectives of the established and already retired athletes (Ronkainen and Ryba, 2020; Scholtz, 2020). Therefore, the purpose of the current study is to explore how strained junior elite performers perceive and negotiate their personal narratives of becoming within a cultural master narrative of being elite performers.

Narrative theory provides new approaches to explore and grasp psychosocial understandings of human lives and phenomena, such as talent development (Carless and Douglas, 2013b, 2017; Martin, 2017). Narrative research is rooted in biographical approaches and allows the study of both structure and agency (McGannon and Smith, 2015; Carless and Douglas, 2017). Viewing the relationship between the self and society as reciprocal and co-constructive, narrative methods offer insights into the process of meaning making of lived experience. Furthermore, narrative research focuses on how experience is situated in time and place and might provide insight into a trajectory of life across time (Carless and Douglas, 2013b, 2017; Ronkainen et al., 2016). Yet few studies within performance psychology have grasped methodology that mirrors this psychosocial interaction and complexity (McGannon and Smith, 2015; Martin, 2017).

Talent identification and development systems may offer social conditions and values that create unique cultures of “eliteness” (Aalten, 2005; Carless and Douglas, 2013b; Rongen et al., 2014). By attending to how the meaning making of junior elite performers’ experiences is shaped within the sociocultural context of TIDS, new knowledge of how performers negotiate their identity, values, and behavior is developed (Echterhoff and Higgins, 2018; van Vianen, 2018; Ronkainen and Ryba, 2020). Noteworthy is the negotiation process that takes part within those narratives that have become a “master narrative” (i.e., culturally dominant and widely told and retold), such as “the performance narrative” identified in the elite context (Carless and Douglas, 2013b; Rongen et al., 2014; Scholtz, 2020). In this study, we were interested in how cultural master narratives within TIDS might influence, override, and silence alternative stories. In turn, this influence might make the co-construction of one’s personal narrative restricted and challenging, perhaps out of line with one’s true and positive sense of self (Carless and Douglas, 2012, 2013b; Hansen and Jessop, 2017).

Traditionally, excellence has been conceptualized in terms of normative performance outcomes, typically medals and world records in sports, and being a top international soloist performer within the performing arts (Miller and Kerr, 2002; Stabell, 2018; Haraldsen et al., 2020). The “performance narrative” (Carless and Douglas, 2013b) is rooted in this tradition and promotes the storyline of preferred identities (i.e., the 24/7 elite performer), expected behaviors (i.e., dedication, passion, and mental toughness), and assumed developmental trajectories (i.e., a linear road to success; Carless and Douglas, 2013b; Kerr and Stirling, 2017; Stabell, 2018). Conversely, substantial concerns have been raised toward the singular performance identity that seems to develop within elite performers (Aalten, 2005; Willard and Lavallee, 2016; Stabell, 2018). TIDS often organize the entire lives of their performers (Aalten, 2005; Christensen and Sørensen, 2009), resulting in exclusion from other activities, relationships, and life goals (Christensen and Sørensen, 2009; Douglas and Carless, 2009; Willard and Lavallee, 2016). Furthermore, scholars have criticized the concept of “mental toughness” (i.e., have self-confidence, manage emotions efficiently, and tolerate pain and pressure) as promoting unhealthy values and risky behaviors (Gabbett et al., 2016; Blevins et al., 2020).

However, in the wake of critical views on “the performance narrative” (Carless and Douglas, 2013b; Rongen et al., 2014; Kerr and Stirling, 2017), a “holistic alternative” has been put forward. In this view, performance development and personal development coexist and require each other; that is, to perform well, you must be well (Miller and Kerr, 2002; Houltberg et al., 2018; Preston and Fraser-Thomas, 2018). In this narrative, the concept of “thriving” is at the core (Brown et al., 2018; Preston and Fraser-Thomas, 2018). Thriving is linked to positive functioning and is described as the dual experience of performance success and well-being (Brown et al., 2018). Hence, the road to dual thriving (i.e., integrating performance and personal development) involves alternative narratives and conceptualizations. For instance, to understand “success” not only as experiencing a linear road to success and being the best but also as being balanced, displaying agency and feeling good (Miller and Kerr, 2002; Wylleman and Rosier, 2016).

Narrative theory postulates that mental well-being “requires a degree of *narrative alignment* between one’s experiences, the stories one tells, and the narrative types available within one’s culture” (Douglas and Carless, 2009, p. 215). This idea is based on the concept of person-environment fit (PE fit), suggesting that people have an innate need to fit with their environments and share reality with others (Amiot et al., 2006; Echterhoff and Higgins, 2018; van Vianen, 2018). Narrative research has proposed that environments supporting performers exploring and “re-storying” personal authentic identity and offering alternative narratives and identities might be beneficial to reducing unhealthy narrative tensions in the PE fit process (Carless and Douglas, 2013b; Willard and Lavallee, 2016; Wilt et al., 2019). Indeed, previous studies have shown that junior elite performers are likely to adapt, instead of resist, to the environment to avoid impeding their social position or their relationships with their teachers or coaches (Stabell, 2018; Haraldsen et al., 2019, 2020). In another study by Kimball (2007) that investigated why collegiate student-athletes subjected themselves to an environment constantly limiting their autonomy, findings indicated that the student-athletes made an autonomous choice to commit to a restrictive lifestyle and, therefore, often accepted the regime. However, they compensated by reframing the experience (i.e., as preparing for the future or being a learning experience) to retain a feeling of agency and coherence (Kimball, 2007). Hence, junior elite performers might experience difficulties in achieving narrative alignment (i.e., PE fit) and, in turn, a harmonious internalization of one’s true self (Amiot et al., 2006; Douglas and Carless, 2009; Echterhoff and Higgins, 2018).

Therefore, the current study set out to explore how strained elite junior performers within TIDS in sports and performing arts perceive and negotiate their personal narratives of becoming within a cultural master narrative of being elite performers. The following research questions guided our work: (a) How do junior elite performers perceive and experience the performance narrative within their TIDS; (b) How do junior elite performers negotiate their personal narrative of becoming elite performers within a cultural master narrative of being; and (c) How does the quality of PE fit, characterized by narrative alignment or tensions, relate to junior elite performers’ perceptions of identity, agency, and physical and mental health.

## Materials and Methods

### Philosophical Assumptions and Research Design

The present study is positioned within the social constructivism paradigm (Denzin and Lincoln, 2011; Creswell, 2013), which focuses on how world views are individual specific and created through a combination of interactions with cultural norms and values, as well as the meanings that individuals attribute to such interactions (Denzin and Lincoln, 2011; Creswell, 2013; Martin, 2017). Thus, we employed a qualitative interview study and narrative design to address the research question (Grysman and Mansfield, 2017; Willis, 2019). Narratives allow research findings to be presented in a way that acknowledges the complexities of unique individual experiences while drawing out a more generalized understanding and knowledge base (Creswell, 2013; Grysman and Mansfield, 2017).

### Participants, Ethical Considerations, and Procedures

We purposefully recruited eight participants (*M* age = 17.31, *SD* age = 0.90) from a previously published cross-sectional study (Haraldsen et al., 2019) who demonstrated more relatively (< 1SD above the mean) sub-optimal functioning (i.e., the frustration of the basic psychological needs of autonomy, competence, and relatedness) than their junior elite peers (Ryan and Deci, 2017; Haraldsen et al., 2019). The frustration of autonomy reflects acting out of external pressure or indirect manipulation. Experiencing failure, stagnation, and inferiority echoes competence frustration, whereas frustration of the need for relatedness is linked to feelings of isolation and socially distance (Ryan and Deci, 2017). They were recruited and interviewed during their subsequent school year. The performers came from prestigious TIDS in sports (*n* = 4; rowing, alpine skiing, and swimming) and performing arts (*n* = 4; music and ballet conservatories). They passed extensive auditions and had 9.56 years (*SD* = 3.21) of previous experience in deliberate practice, on average.

The study was carried out after securing ethical approval from the state-run Norwegian Center for Research Data (Ref: 55635 EPA). Access was gained through dialog with the sports federations and TIDS leaders. The first author, who had extensive lived experience with TIDS in dance (i.e., as a youth performer, teacher, and teacher educator), contacted and recruited participants directly through email and a phone. The participants received oral and written information about the study and its procedures, ethical concerns, and anonymity.

The first author conducted the face-to-face interviews in suitable locations of the participants’ choosing. As the interviews were comprehensive and long-lasting, they were divided into two parts, but some of the questions were repeated in both interviews to connect the interviews together. The data collection strategy was partly due to difficulties in accessing the elite junior performers, because of busy training and competition time scheduling and long travel distance. Also, in addition to this interview study, a longitudinal and more comprehensive quantitative study was conducted at the same time. As the quantitative study did comprise several of the same issues and topics, the two studies informed each other, and the first author traveled to meet with the junior elite performers and their talent development programs several times during the data collection year, enhancing indirectly the data. The interviews were audio recorded (ranging from 66 to 154 min each) and transcribed. We used NVivo 11 to facilitate the first part of the analysis process (i.e., coding and thematic analysis).

### Data Collection

We used semi-structured interviews that were thematically structured and inspired by the research questions. The interview guide focused on four main perspectives: (a) perception of identity (personality, ability, talent, dedication, goals and ambitions, and motivation); (b) perception of the elite performance context (learning environment, coaching/teaching style, and interrelations in the TIDS); (c) perception of becoming (linking past, present, and future, the performance development curve, important events, incidents, and turning points); and (d) perception of well-being (i.e., self-worth, emotional states, vitality/exhaustion, performance anxiety, injuries, and life satisfaction). In line with the narrative approach, the participants were asked broad and open-ended questions within each topic, providing a fair degree of freedom of what to talk about and how to present their narratives (Grysman and Mansfield, 2017). Additionally, follow-up questions were used to dig deeper into core perceptions and experiences that appeared during the interviews.

### Data Interpretation and Analysis

**Figure 1:**
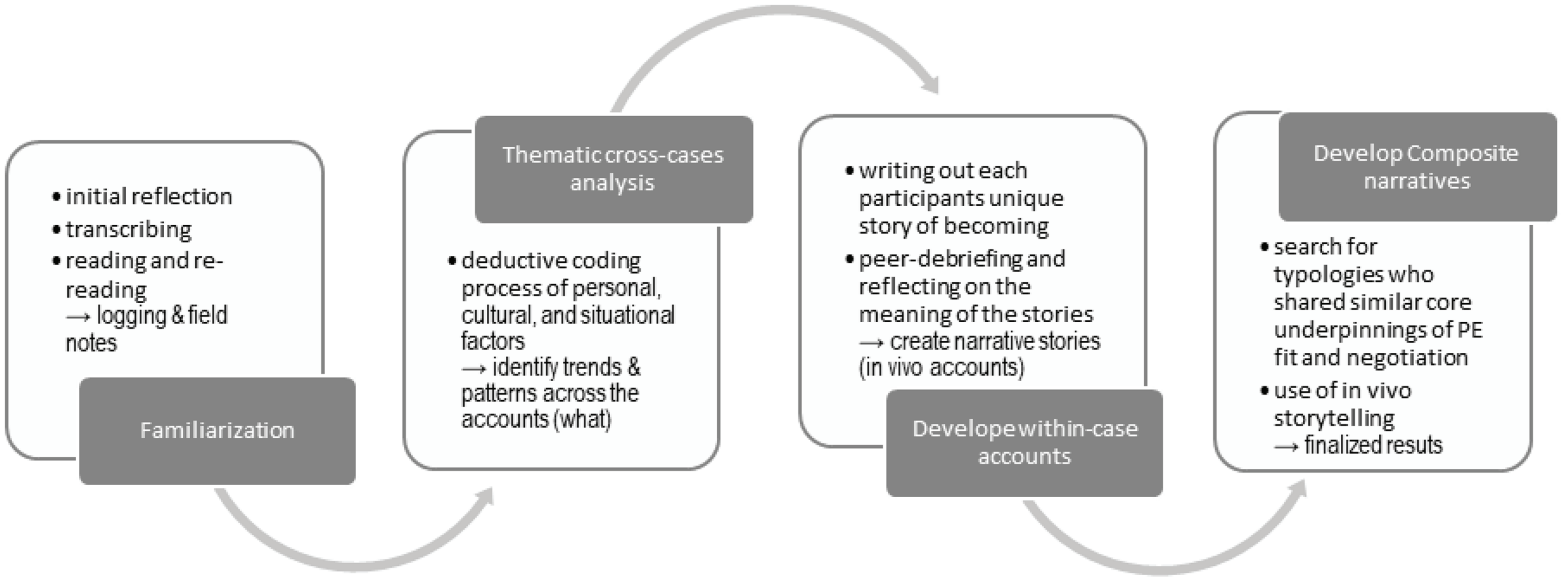
The process of data analysis.

We first familiarized ourselves with the data through observing, reflecting, and intuitively experiencing the data (Figure 1). Familiarization occurred immediately after the interviews and during the process of initial reflections, transcribing, and rereading the accounts, and is part of the field notes. After the initial familiarization, we conducted the narrative analysis in three stages: First, we focused on a thematic cross-cases analysis, categorizing specific themes, typologies, plots, and structure (i.e., particularly personal, cultural, and situational factors) in the narratives (Riessman, 2008; Smith and Sparkes, 2009; Willis, 2019). Specifically, we focused on (a) how the performers understood and executed their identity; (b) perceptions of the elite performance context; (c) perceptions of becoming (i.e., trajectories and important shaping relationships, incidents, and events); and (d) perceptions of experienced well- or ill-being. We used a deductive three-step coding process: (1) clustering meaning units of text, extracted into each predetermined theme (themes a–d); (2) categorization within themes; and (3) synthesis and analysis across themes and categories. Hence, this procedure allowed us to identify trends and patterns in themes and issues apparent across the participants’ accounts.

Next, we developed within-case stories by focusing on the performers’ ways of negotiating the complexity of becoming elite performers within a domain-specific performance culture. We focused on narrative tensions and alignment (i.e., the role of PE fit). Assisting this analysis process, we adopted the role as a “storyteller” (Smith and Sparkes, 2009) and wrote out each interpreted story of becoming a narrative novel and then peer debriefed it (i.e., trajectory, main characters, important turning points and events, and underlying culture). We considered this to be an important analytical tool, showing rather than telling, using creative nonfiction to represent the research findings without making things up (Cavallerio et al., 2016). We asked questions about the data (Riessman, 2008), such as: (a) Why and for whom are the stories told in this way? And for what purpose? (b) What cultural norms and values are the stories embedded in? And what is taken for granted? and (c) Are there gaps or inconsistencies revealing alternative or counter-narratives? Hence, this procedure allowed us to identify similarities and differences in themes and issues apparent in each unique participant’s account.

After this process, we were left with a set of complex and personal interview accounts and stories of becoming, engaged in the tricky question of how to present this richness fairly, while maintaining the participants’ anonymity. As Norway is a country of few inhabitants, TIDS comprise small groups and transparent cultures, making anonymity, especially challenging to maintain. We then decided to use composite narratives to present the findings, illustrating the different types of narrative negotiation of becoming an elite performer identified across the different stories (Willis, 2019). Thus, the third step was to search for typologies across the individual narratives of the performers who shared similar core underpinnings of PE fit and negotiation (Smith and Sparkes, 2009; Willis, 2019). This approach, which has been used in other studies (Carless and Douglas, 2013a; Willis, 2019), allowed us to reduce the total complexity and preserve necessary depth in each narrative and, at the same time, ensure anonymity. Different groups of composites were considered, and we chose the final solution, as it seemed the most meaningful representation of the data in relation to the purpose of the present study. To maintain a direct link to the original data and narratives, we decided that all the quotations used would be literal quotations from the individual interviews (Riessman, 2008; Willis, 2019). In this process, we continued with the storyteller approach. In developing the stories, we used not only the key themes from the analysis but also the participants’ own words from the interviews. By offering these embodied “*in vivo*” accounts of human lives, we believe that the results might reach multiple audiences, not only the academic, but also likely to produce a higher applied impact (Smith et al., 2015).

### Quality and Rigor

Acknowledging recent discussions regarding rigor and quality in qualitative and narrative research (McGannon et al., 2019), we view quality criteria as values and actions that enhance quality through bringing transparency and trustworthiness into the research process (Smith and Sparkes, 2009). In line with the narrative approach, establishing the authenticity of the participants’ accounts became essential (Nowell et al., 2017). Hence, as asymmetric power relations are present in all research with humans, steps were taken to safeguard the participants in order to elicit reflections that were as open-minded as possible. For instance, the interviewer prepared for the role of a facilitator, active listener, and supportive audience, and, additionally, applied a warm-up session to safeguard the participant (e.g., Brinkmann and Kvale, 2008; Morrow, 2008). Next, member reflections were gathered in “after-talk” conversations about the interview and topic discussed after the “formal” interview was conducted, and the participants were encouraged to make contact if new reflections, thoughts, or insights appeared later. However, as the participants did share reflections and opinions more freely in the “after talk,” none of them contacted the researcher afterward. Moreover, when interpreting the accounts (i.e., data analysis), the first author’s own long-term, lived experience was used actively as an asset to enhance contextualization, familiarization of implicit culture and language use, and access to deeper layers and nuances of the participants’ lived experiences. We also used exemplifying verbatim quotes in the results section to increase authenticity (Nowell et al., 2017).

To enhance trustworthiness, we used the concept of coherence, which focuses on the justification of proposed knowledge claims in qualitative research (Smith and Sparkes, 2009; Smith and McGannon, 2018). Proactive planning, prolonged engagement (i.e., a 24-month-long research process), ethical considerations, collaborations with applied fields (i.e., meetings, lectures, seminars, and teacher/coach workshops), and extensive use of reflexivity and peer-debriefing sessions were all the strategies that we used to ensure coherence.

Reflexivity is a research tool that addresses the researcher’s role as an active component in the research process (Finlay, 2002). Thus, reflexivity has assisted our process of monitoring, coping with, and making transparent our role as researchers in the research process (i.e., access, interview process, analysis, and interpretation; Berger, 2015). To address and deal with this positionality, and to develop plausible interpretations, the first author applied the use of a reflective log, which was meta-reflected and peer debriefed (Smith and Sparkes, 2009; McGannon et al., 2019). The peer debrief sessions were useful and reflected how the interviewer’s and the researchers’ different experiences and positions toward the study objects affected the data collection and analysis process. As a result, this tool helped with becoming aware of a plan and deals more directly with issues linked to the co-constitutive nature of data generation and to really dig deep into the process of interpretation and meaning making of the participants’ accounts in order to sort out the more generalized perspectives.

## Results

We present the results in two parts. We start by presenting the junior elite performers’ general perceptions of the culturally dominant performance narrative in the TIDS. Next, we present the three different composite narratives of negotiation within the overarching master narrative: “Striving to stay at the top of the game” – obsessive and externally driven alignment, “Just hanging in there” – tensions, and “When the going gets tough” – disruptions from alignment (see Table 1). In the presentation of the composite narratives, we focus on the role of the junior elite performers’ self-perceptions of their performance identity, the negotiation process, and the PE fit, as well as the experienced consequences (i.e., identity challenges and well-being). Direct quotations are marked by number and domain (a = art, s = sport). To retain anonymity, gender and activity are masked, and “(s)he” and “him/her” are used as subjective and objective pronouns, respectively.

**Table 1:** Overview of the three composite narratives.

Identity perceptions	Negotiation, PE-fit	Thriving
← Past →	← Present →	← future →
**Composite narrative: Striving to stay at the top of the game**		
“I have what it takes” PE alignment: Early identified talent, dedicated, high-achieving, self-confident, and competitive	“I am very good at dedicating myself continuously to hard work.” PE-alignment: Living out the performance narrative, being at the top of the game; however, in an obsessive, external driven, and ambivalent way underpinned by conditional motivation, perfectionistic tendencies, and fear of failure	“I like the feeling of progression, of mastery, and success.” Mostly positive thriving: Flow experience, achievements, meaningful and rich life Aspects of risk behavior and ill-being: Stress and performance pressure, performance anxiety, emotional distress, doubts, overtraining, and signs of burnout
**Composite narrative: Just hanging in there**		
“The characteristics that I hold are atypical of an elite performer.” PE misfit: Externally shaped and coerced identity, ambivalence, lack of self-confidence, and doubts	“I feel like I have developed against the odds.” Narrative tensions: Constantly striving and still feeling failure in living up to expected standards in the performance narrative. Underpinned by lack of inner drive, conditional motivation, perfectionistic concerns, lack of coping strategies, and a plan B	“Even though the activity drains my energy, it allows me to express myself.” Mostly aspects of ill-being: Identity confusion, inauthenticity, lack of motivation and joy, emotional distress, performance anxiety, exhaustion, and burnout, Positive thriving: Involved in artistic processes, deeper living, expressing ideas.
**Composite narrative: When the going gets tough**		
“I strive to become that successful performer.” PE-alignment: Early identified talent, dedicated, high-achieving, self-confident, and competitive	“My development curve has been going upward all the time, until I got injured.” Disruption of narrative alignment: When faced with injuries and sickness, identity crisis occurs. Failing to provide an alternative narrative nurture feeling of shame, humiliation, and frustration. Proactive coping and support help to recover and refocus, whereas performance pressure, loss of status, and attention worsen the crisis.	“It is mentally tough.” Mostly aspects of ill-being: Identity crisis and doubts, loss of meaning and motivation, emotional distress, performance anxiety, exhaustion, burnout, and risk of dropping out. Positive thriving: Refocus, restart after a break, sustained hope and optimism, resilience, and renewed motivation.

### The Performance Narrative: “Talent Factories Aiming for the Top”

#### It Is So Cool to Be an Elite Athlete

The performers are proud and honored to be part of the TIDS. They feel special, talented, and selected, and it boosts their self-confidence: “I found it so cool to be an elite athlete… It makes me feel good. I can prove myself, and I gain self-esteem” (5 s). It is also thrilling to be with other peers who are talented and share the same goals and interests: “It is a very thriving environment. Everybody is interested and dedicated, and we are good friends; it nurtures the feelings of joy and motivation (2a).” As the TIDS consists of small and exclusive groups (*n* < 15), the peer relations are close and mostly supportive: “The national junior team is like a small family. It is a lot of fun but, at the same time, very serious when we compete (5 s).” However, despite being intriguing: “Competition provides a channel for my restlessness and is a source of excitement in my life (5 s),” competitions and striving for success sometimes are also a source of stress and worry, as all the good things seem to depend on how you perform: “Competitions are risky and out of control; there is a lot at stake if you should fail repeatedly. Others will think you are a failure, and you risk getting deselected and excluded from important opportunities (2 k).”

#### It Is a Pressure to Perform Well

The performers tell stories about exclusive and highly professionalized “talent factories,” with a history of producing and showing off excellent performers. The young performers are fully aware of and share the aim of performance excellence, and they know what is at stake: “It is a pressure to perform well… then you got a good reputation, and you get selected for performances and get access to more opportunities (2a).” Reaching goals and accomplishments seem to be more important than the process of becoming: “It is always the time and the result that count, not what you have developed (6 s).” To be among the best, the top group is the most important currency. It may be exchanged with social status among peers: “It is all about respect in my sport culture. The better you are in your activity and more perfect you perform, the more respect you get (8 s),” teachers: “the teachers really like having a good ‘star’ student” (2a), or coaches: “I think it gives the club and the coach status to have a successful athlete” (6 s). Thus, being good means being popular at the activity: “It is very competitive… If you do not perform well, it could be difficult to be socially accepted and included in the social group (2a).”

#### It Is a Very Hierarchical System

The TIDS are well-driven and structured talent development programs. The young performers are often told what to do, how to behave, and how to prioritize: “It is a very system-driven activity. It is planned down to the tiniest detail, and extreme coach-led activity… It is a very hierarchical system… It is the coach that makes the decisions (6 s).” The young performers themselves support this system and find it natural: “Of course it has to be this way… That is why we have teachers so that they can teach us what they already know and show you how to become a professional performer (2a).” The performers also admire and respect the teachers and coaches as authority figures: “My teacher is older and more experienced in the field. Naturally, I respect and listen to her to a greater extent than vice versa (1a).” Hence, asymmetric power distribution is naturally the case: “The teachers have a lot of power. They, basically, can say and get the students to do whatever they want. However, they do not always exploit their position (3a).” Knowing that they might misuse their power, the performers find it best to avoid possible provocations: “We have to adjust to the teachers’ mood, taste, and comfort (3a).” They also have had previous experiences with controlling teachers or coaches: “I know that she puts up a facade to affect me psychologically, to get me to work harder, or to perform better” (4a). As such, to do as told and expected is seemingly the best way to keep the coach satisfied: (my coach is pleased) “when I listen to him… Also, if I do not cause any trouble or oppose but, instead, do as told and follow instructions, or if I reach one of his goals (5 s).”

### Composite Narrative 1: “Striving to Stay at the Top of the Game”

#### I Have What It Takes

S(he) is a typical early identified talent that is accustomed to leaning on natural talent and being successful without expending that much effort: “I had a good start. I had a natural talent and was way beyond my age group (2a).” (S)he expresses much self-confidence: “I think that I am going to make it because I have what it takes. I am not an average person” (1a) and seems mostly to enjoy the process of becoming an elite performer. (S)he fosters motivational drive from success, approval, and social status: “I want to become a star and would like people to think of me with honor and respect” (1a). The relationship with the teacher/coach is personally fulfilling: “My teacher is focused on my development and personally interested in my well-being” (1a), and trusting: “I feel I have a good relationship with my coach; we can talk about everything (8 s).” (S)he has been active for many years and experienced a lot of mastery and success on the way to the top: “My performance curve has been positively steep. I have developed faster than normal and made development leaps (8 s).” Hence, (s)he mainly supports the TIDS’ culture, which seems to correspond well with his/her high ambitions and goals. It has been almost too easy: “I feel that it has been mostly an easy and positive journey (2a),” and (s)he has not yet faced severe adversity or stagnation: “I have never really experienced failure” (1a). Thus, (s)he is really at the top of the game, succeeding and with high social status within a “talent factory,” aiming for the top: “The activity plays an important role in my life. I spend a lot of time on the activity, and I like to practice it. I have close friends, and I feel that it positively affects my quality of life (8 s).”

#### I Am Very Good at Dedicating Myself Continuously to Hard Work

Beneath the ambitious and successful facade, (s)he appears to face some challenges while striving to maintain his/her top position. (S)he struggles with high expectations and pressure to perform well, to progress all the time: “I started to practice and work really hard because my teacher threatened to quit if I did not live up to his expectations (2a).” This seems to nurture some innate perfectionistic tendencies: “I need to feel in control of my performances… I practice repeatedly until I reach a feeling of perfection and control” (1a) and external motivation, conditional on whether success, or failure is realized: “Motivation is hard to control: If I perform well, I get motivated… If I fail, then I get upset and disappointed, then I lose motivation (8 s).” In turn, the constant strive to maintain the top position nurtures a kind of obsessiveness: “I get stressed out and get into obsessive periods when I fall behind” (2a) and emotional distress when mistakes are made: “[When I make mistakes], I get emotionally dark, it feels heavy, and I get angry and disappointed in myself (8 s).” In sum, (s)he, constantly, must work hard and remain dedicated: “My perfectionism has made me very self-disciplined. I am very good at dedicating myself continuously to hard work, to keep up with the routine… make the best out of each opportunity (1a).” However, all the hard work and dedication feel exhaustive and, sometimes, too narrow-minded: “I get drained of energy and must down-prioritize other things (2a).” Hence, (s)he is on the edge of being demotivated, questioning if it is worthwhile: “I have doubts about my activity… Sometimes, I question whether I really want to become an elite performer (2a).”

#### I Like the Feeling of Progression, Mastery, and Success

(S)he feels ambivalent about the activity: “Even though there are a lot of positive experiences, I feel very exhausted from all the hard work (1a).” Yet, (s)he mostly finds the hard work satisfying and worthwhile: “I like the feeling of progression, of mastery, and of success, both the small and the great accomplishments… and I feel that I have developed self-control, dedication, and endurance (2a).” It is like a state of flow, of being deeply dedicated and involved in the learning process: “My activity has added more color and motivation to my life; I experience this as a more meaningful life (1a).” However, holding such a top position comes with a price. (S)he constantly fears failure: “I get very anxious during a competition… I feel sick and numb, then becomes stressed that the anxiety might disrupt my performance (8 s).” (S)he also fears losing the advantageous social position that (s)he has achieved: “I experience performance anxiety each time I have to perform… It is embarrassing to fail… especially when playing in front of peers because it might affect my social status and opportunities (2a).” When faced with pressure, (s)he uses different kinds of coping strategies, such as positive self-talk: “I try to think that I am in control and that this is something I have practiced a lot, I am brilliant, and I can easily nail this” (1a). However, previously, when (s)he was younger, (s)she did not know how to deal with the pressurized situation: “For a long time, I avoided competitions completely; now, I try to distance myself from the emotions and focus on body awareness and breathing (2a).” (S)he does also try to learn from his/her mistakes: “First, I get irritated and frustrated, but, soon, I just go and work on it… find out how I can solve the problems and improve myself (1a).” It helps a lot that (s)he receives support from others, such as the teachers/coaches, family, and peers: “I get, instantly, often balanced and engaging feedback… This is, indeed, the way I progress (1a).” Altogether, (s)he balances on a knife’s edge between peaking and crashing, loving and hating the activity at the same time: “The activity has given me an extra dimension in life; it makes my life richer (1a)”; “Sometimes, when I am in a straining period and receive bad results, I experience a lot of negative emotions and might even start to reconsider my future career for a while (8 s).”

### Composite Narrative 2: “Just Hanging in There”

#### The Characteristics That I Hold Are Atypical of an Elite Performer

(S)he is a performer who operates in the shadows of typical “star students”: “I do not have the greatest potential. I feel like I have developed against the odds (3a).” (S)he says that (s)he deviates from the norm and identity of an elite performer: “The characteristics that I hold are atypical of an elite performer: I am easily bored, I am not structured, I do not like to self-practice, and I find that practicing the activity is boring and uninteresting (4a).” (S)he is just hanging in there, lacking a clear purpose, inner drive, and enjoyment. (S)he participates because her parents expect it: “I perform because I come from an artistic family… If I could choose again, I would probably have chosen something else (4a).” (S)he feels that (s)he rarely lives up to the expected standards: “I seldom appreciate my achievements (3a),” and never get that good feeling: “No matter what I accomplish, I never feel satisfied (3a).” The training has become more of a daily routine, a natural part of life: “My parents placed me into a studio, and, after a while, I started to appreciate it – it was fun. Then I just have continued. Now, it is a huge part of my life (3a),” (S)he does not have a “plan B” and has not prioritized an academic identity to lean on: “Even though I have been really demotivated in some periods, I fancy no other alternatives (3a).”

#### I Feel That I Have Developed Against the Odds

The development process feels a bit hard, working often against the odds and facing different challenges. Thus, (s)he struggles to find the inner drive and a stable motivation: “My motivation is very unstable: Suddenly, I get inspired and work well. The next day, my motivation is completely lacking. Generally, I struggle to work hard over long periods (4a).” (S)he has experienced many bumps and detours on the road to success over the years: “It has been different periods with ups and downs (4a),” and it has been hard: “It has been lots of flounders… Overall, I feel that I have developed against the odds (3a).”

Another issue is that (s)he seldom has a general feeling of mastery and success; instead, the most prominent feeling is that of failure and concern: “I seldom experience feelings of mastery. There is always something that is not perfect to improve, and, if only a tiny thing goes wrong, I often feel that everything falls apart (3a).” Hence, (s)he is often faced with stress and fear related to performances: “I get very nervous when I am about to perform… The anxiety transfers to my body. I get tense and stiff in my upper body, and it negatively affects my performances (3a).” A problem is that, when (s)he is faced with these challenging performance situations, (s)he does not really know how to handle the stress and fear. (S)he finds it a bit difficult that his/her main teacher/coach is not very helpful and does not seem to care about the troublesome stuff and only appreciates his/her success. Yet, (s)he thinks that (s)he is the one to blame: “If I do not do what is expected of me and work hard, I do not deserve such a brilliant teacher, or positive attention and approval (4a).” However, (s)he hopes that it nurtures development in the end: “If the teacher praises you all the time, you will never improve yourself or grow (4a).” Hence, (s)he often focuses on avoiding stressful situations: “I try to avoid making mistakes and not look like an idiot (4a).” Over the years, when facing performance situations, (s)he has developed a way of thinking and behaving where (s)he leaves the body behind, distancing from the fearful body: “I think my brain disconnects, and I think, ‘What if I just erase this situation and ‘sleep’ it through,’ so that you do not make mistakes and look like a complete idiot (4a).”

Despite challenges and setbacks, (s)he feels that the performance curve has been positive in the long run: “I started at the bottom and have worked myself upward (3a).” (S)he admits that (s)he could have worked harder and that (s)he has spoiled some opportunities: “I have not really made that much of an effort over the years (4a).” However, (s)he, recently, has started to take the activity a bit more seriously, heading for a professional career: “The last 2 years, I have shifted my focus and improved the quality of my practice, even though I must admit that I do not invest that much in quantity (4a).”

#### Even Though the Activity Drains My Energy, It Also Allows Me to Express Myself

(S)he often feels burned out and drained: “I feel exhausted all the time. Often, when I am exhausted, I feel numb. I do not have the energy to do anything outside the activity (3a).” (S)he has also faced some injuries on the way, and a new injury is a thing (s)he fears the most: “When faced with an injury, you get a huge setback, and it is so hard to get back on track, and you feel so terrible and blue… I fear injuries and I, sometimes, worry about becoming reinjured (3a).”

Despite challenges and negative aspects, (s)he notes many positive elements that somewhat balance the negative. For instance, the fulfilling sensation of mastering difficult skills and being involved in artistic processes: “When I perform, not at auditions or competitions, but in a show in front of an ordinary audience, then I feel happy. I can feel afterward that I am cheerful inside (3a).” Also, being involved in creative processes is something (s)he finds meaningful and worthwhile: “Even though the activity drains my energy, it also allows me to express myself and my ideas… new and creative ways of expressing interesting ideas through the art add energy (4a).”

### Composite Narrative 3: “When the Going Gets Tough”

#### I Strive to Become the Successful Performer

(S)he is ambitious and self-confident. (S)he has always been among the best since childhood and has a leading position in the activity group: “My self-esteem is very good. When I was younger, I was probably perceived as being cocky. I was not cocky as a person, but I had very good results (6 s).” Today, (s)he works hard and makes choices according to what (s)he knows is expected of an elite performer: “It is part of who I am; it has always been. It defines who I am, what I can do, and how I prioritize things (6 s).” Even though it is difficult, (s)he appreciates this way of living and the status that follows: “I feel more important when I perform well; others do not appreciate me in the same way when I perform poorly; hence, I strive to become the successful performer, and I believe I have what it takes (6 s).” Motivation is not an issue. (S)he has always been and still is highly motivated: “I love doing the activity; it has always been my thing… I get a magic feeling (6 s).” However, (s)he is somewhat addicted to the identity and status that follows: “I feel more important when I perform well (5 s).” Thus, (s)he sometimes feels pressured to practice and keep up the success, and fears losing the leading position: “I feel that others do not appreciate me when I fail. The better I perform, the more I am appreciated by others (6 s).” (S)he has been in a competitive environment for years. (S)he likes it this way; it matches the way (s)he focuses on the development and helps in reaching his/her ambitious goals: “I focus on reaching good results, beating personal records, and defeating new competitors (7 s).”

#### My Development Curve Had Been Going Upward All the Time, Until I Got Injured

Competence is what matters in the training milieu; hence, the success (s)he has experienced has been profitable: “Last season, when I performed very well, the coaches were on me all the time with attention and interest (7 s).” Lately, things have changed, and (s)he is not sure of his/her own position or outlook anymore: “This season, when I have been sick and not able to produce good results, the coaches are indifferent. The training is all up to me, and, sometimes, I must, instead, coach younger performers (7 s).” Looking back on the good old days of thriving and being at the top of the game for years, (s)he could never have imagined how things could be: “My development curve has been going upward all the time, until I got injured. I have been through a tough year. I got injured, got chronically ill, was exhausted, and performed poorly (6 s).” These days, things are really hard, and (s)he feels that (s)he is trapped in a bad cycle, like dominoes about to fall: “After the injury, I fell into a bad cycle of being injured, sick, and demotivated (6 s).”

Even though (s)he appreciates the TIDS, (s)he finds it a bit controlling and limiting. The coaches do not always listen to what (s)he, as a performer, really needs: “The system is trumping the individual’s needs (7 s).” When (s)he is now experiencing difficulties, (s)he wishes that the coaches could have been more caring and sympathetic: “I wish that the relationship was more personal and not so distant (6 s).” This makes it difficult to be really honest and share inner worries, fears, and negative thoughts that (s)he is experiencing during these tough times: “The relationship with my coach is not working. It is difficult to communicate, it is not very close, and I feel that it is difficult to state my own opinions and feelings (7 s).” Instead, (s)he remembers a former coach who coached him/her some years ago: “My coach was tuned into me… He invested in the relationship and did not yell at my mistakes but, instead, asked questions (5 s).” These days, (s)he needs such a coach: “He was like a friend. I could talk to him about anything, and we often laughed together” (5 s). Fortunately, (s)he has a family that offers support and care: “My family knows nothing about the details of my activity, and they do not pressure me at all. They only provide me with support, praise, and positive feedback. I feel that I have a safe haven at home (5 s).”

#### It Is Mentally Tough

Being a junior elite performer these days is different from when (s)he held a top position, and (s)he sometimes feels a bit ambivalent about the activity and has started to doubt whether this is the right path for him/her: “It is mentally tough (6 s).” (S)he thinks that this period of adversity and stagnation has been quite challenging: “I get disappointed if I do not live up to the high expectations, and then I think: “This is not me.”… I get disappointed and frustrated… I feel ashamed and embarrassed (5 s).” The situation sometimes seems impossible to overcome, and then (s)he loses motivation and focus: “I get indifferent when I am injured and feel that I am faced with a challenge that is impossible for me to overcome (5 s).” (S)he feels mentally fragile as well: “I get mentally weak, and my body feels numb and heavy (5 s).” However, quitting is not an option: “The activity is an important part of me. I could not just quit. It would be as drastic as moving to Africa and leaving my family behind (7 s).” Thinking of the good old days and all that (s)he has accomplished is helping to mentally turn around toward a positive way of thinking: “I have what it takes to succeed. I have the technical and tactical understanding and, maybe, a top performer if I am willing… and I am mentally strong. I seldom give in (5 s).” (S)he also knows that (s)he is good at handling things and has many strategies at hand: “I have routines for eating, sleeping, and training, and I work a lot on trying to stay positive and happy, to not be mentally dragged down (5 s).” (S)he just must focus on the positive things because there are still many positive experiences: “I still have happy days. They are important, as they make me feel good, strong, and alert (5 s).” (S)he also enjoys writing in a diary: “I use a reflective log as a tool. I write a lot to clear my mind, especially before sleeping (5 s).” Today, (s)he feels a lot more optimistic. The long period of absence from the activity has been helpful, as (s)he was able to distance himself/herself from it all and examine the activity from another perspective; (s)he is now in the process of reset: “I came back and just competed for fun. I suddenly performed outstandingly and set new records in all the competitions. I rediscovered the good feeling of flow (6 s).” Indeed, (s)he is still feeling grateful, hopeful, and self-confident, believing that (s)he, 1 day, will make it.

## Discussion

The present study’s purpose was to explore narrative tensions in strained junior elite performers’ experiences of becoming elite performers. In line with the outlined research questions, we start by reflecting on the performers’ perceptions of the dominant narrative within their TIDS. Next, we focus on the PE fit and how the junior elite performers negotiated their personal narratives of becoming elite performers within their TIDS culture. Lastly, we reflect on how narrative alignment or tensions relate to junior elite performers’ perceptions of identity, agency, and physical and mental health.

The findings identified performance-focused and professionalized TIDS, hence documenting the “performance narrative” as being most dominant in these elite cultures (Carless and Douglas, 2012, 2013b). The TIDS were literally described by some performers as “talent factories,” leaving an impression of the young performers as being objectified as capital to invest in to reach charted performance goals (Carless and Douglas, 2013a; Kerr and Stirling, 2017). Hence, these characterizations are at odds with the “holistic narrative” that postulates student-centered and ecological perspectives on talent development (Christensen and Sørensen, 2009; Jowett et al., 2017), alongside the need for integrating personal and performance development (Miller and Kerr, 2002; Strachan et al., 2016). Consequently, the norms and values for what constituted “eliteness” became rather narrowed and limited.

Junior elite performers are in the late process of identity development, and, hence, they have relied and still rely on the socialized narratives that circulate within their performance culture to direct their interpretations (Carless and Douglas, 2013b; Ronkainen and Ryba, 2020). The findings showed that these cultural narratives were presented by authority figures, such as teachers/coaches, role models, more skilled peers, and/or the media. Aligned with the “performance narrative,” they suggested which past – being an identified talent with early success, present – being a 24/7 dedicated, successful, and happy performer, and future – being a thriving international top performer was the most desirable (Ronkainen et al., 2016; Ronkainen and Ryba, 2020). Indeed, the “performance narrative” was echoed in the way the junior elite performers described their ideal identity, their “dreams,” and the road map to what constituted “success” (Carless and Douglas, 2012, 2013b). As a paradox, the results revealed that the limited perception of “success” as winning and being a top performer reduced the participants’ perceptions of their own success. Yet, implicitly, the junior elite performers told of several indicators of success, which contributed to outbalancing the scale, making an elite performer, occasionally, both meaningful and worthwhile. For instance, there was a “social narrative” of belonging and being part of an in-group of like-minded peers and teachers/coaches that shared the same goals, interests, experiences, and identity categories (Carless and Douglas, 2012; Ronkainen and Ryba, 2020). Furthermore, we observed a “flow narrative,” of deep and internally driven involvement, learning, growth, and, thereby, meaningful self-realization (Carless and Douglas, 2012, 2013b; Orta et al., 2017). Lastly, a “lifestyle narrative,” reflected in a way of living that is healthy, linked to good values and fosters advantages attributes in life, was apparent (Ronkainen and Ryba, 2020). Altogether, these alternative narratives, which were told as parallel side stories by the performers, represent potential narrative resources to draw on for future TIDS in adapting a more holistic approach (Miller and Kerr, 2002; Strachan et al., 2016). They all represent legitimate ways of becoming elite performers and might be an inspiration for narrative diversity in TIDS (Carless and Douglas, 2013b; Ronkainen and Ryba, 2020).

The three composite narratives demonstrated different ways of relating to the dominant “performance narrative” in the TIDS and, hence, different examples of PE fit (Fulmer et al., 2010; van Vianen, 2018). The “Striving to Stay at the Top of the Game” typically demonstrated a PE alignment. These junior elite performers believed in and tried to live out the story of “the performance narrative” (Carless and Douglas, 2013b). The match was somewhat successful, as their stories became confirmed and supported within the TIDS, and they did express being at the top of the game, highly contented and motivated. Still, they experienced some tensions and implicit costs. As they tried to be or become something externally defined and expected, their alignment could also be interpreted as external and coerced, an identity they fitted into like a “costume,” more than an identity derived from an internal agency and based on their true selves (Scholtz, 2020). This interpretation shares similarities with the concept of “performance-based” identity (Houltberg et al., 2018). Performance-based identity is shown to be associated with high levels of perfectionism, conditional self-worth, and fear of failure, alongside low levels of purpose, global self-worth, and life satisfaction (Houltberg et al., 2018). By contrast, Houltberg et al. (2018) found a “purpose-based” identity profile of elite athletes that included high levels of purpose, global self-worth, and a positive view of future life. For purpose-based performers, there are important to experience meaning and agency in their process of becoming. The junior elite performers that are represented in the “Striving to Stay at the Top of the Game” composite narrative share, in our view, characteristics with a performance-based identity profile and represent a PE fit that has external and obsessive features (Houltberg et al., 2018; Wilt et al., 2019; Scholtz, 2020). Hence, despite a good PE fit, this composite narrative represents implicit (i.e., concealed to the performers themselves) underlying ambivalence and narrative tensions.

The second composite narrative, “Just Hanging in There,” represented narrative tensions and a PE-misfit (Carless and Douglas, 2013b). As they literally stated, these junior elite performers did view themselves as “atypical elite performers, developing against the odds.” By stating this, they admitted that there exists a “typical” way of being an elite performer and a “right” or “wrong” way of developing as an elite performer. Unfortunately, they did not resist this “typical” perception of becoming an elite performer, as previous studies have identified it as one way of coping with narrative tensions (Carless and Douglas, 2013b). Resisting and re-storying the narrative would have been a more self-determined and healthy way of coping with the PE-tensions (Houltberg et al., 2018; Scholtz, 2020). Instead, their performance identity seemed to have been externally shaped since childhood, first, by their parents’ expectations and, later, by the TIDS’s performance culture. As a result, they lacked the self-esteem and agency to resist the cultural narrative and re-story their identity. After years of trying to fit in and to live up to other expectations, they were “just hanging in there,” and seemingly lacked purpose and ownership in their own development process (Houltberg et al., 2018; Wilt et al., 2019).

The last composite narrative, “When the Going Gets Tough,” demonstrated disruption of narrative alignment. From being at the top of the game and successfully living out the storyline of the “performance narrative,” they experienced an identity crisis during a period of injury or sickness. Scholars have suggested that reliance on a single dominant narrative might be difficult when performers experience no longer fitting the master narrative (Smith and Sparkes, 2005; Douglas and Carless, 2009), which is echoed in our findings. These experiences are linked to the concept of “narrative wreckage” (Douglas and Carless, 2009), a concept describing performers who experience a PE-misfit and fail to provide alternative narrative templates, hence losing the sense of meaning and unity in life (Douglas and Carless, 2009). The junior elite performers in the third composite narrative seemed to experience “narrative wreckage,” expressed by feelings of failure, shame, and humiliation, which, in turn, escalated into a paralyzing identity crisis (Douglas and Carless, 2009; Rongen et al., 2014). Also, the role of the TIDS environment (i.e., offering support, flexibility, and hope vs. reject, deselect, and devalue) became a central theme in this third composite narrative. The findings revealed that the teachers or coaches possessed much power, which they used in both positive and negative ways. However, in line with previous research (Carless and Douglas, 2013b; Kerr and Stirling, 2017), the TIDS themselves did also seem “trapped” within the dominating “performance narrative” and prioritized performance over personal development when they conflicted. As a result, the TIDS ended up nurturing and maintaining the performers’ vulnerability and narrow identity, which, in turn, increased risk behavior and compromised performers’ physical and mental health (Rongen et al., 2014; Houltberg et al., 2018; Blevins et al., 2020).

Despite the different PE fit, the strained participants who operated within the “performance narrative” seemed to experience increased risk. The performers in the “Striving to Stay at the Top of the Game” composite narrative did seemingly experience narrative alignment and reported many positive experiences of thriving. However, as their identity, motivation, positive emotions, and social status were contingent on sustained success, there was evidence of risk behavior. This was played out as perfectionistic tendencies, overtraining, overload, and performance anxiety, which, as such, placed them in a storyline that balanced on a knife edge between peaking and crashing (Rongen et al., 2014; Gabbett et al., 2016). In the long run, these performers could easily end up in the third composite narrative, “When the Going Gets Tough,” if faced with a period of injury or sickness (Smith and Sparkes, 2005; Douglas and Carless, 2009). The first and third composite narratives shared clear similarities in the way the performers had lived out the storyline of the “performance narrative” in the past. However, the third composite narrative represented stories of disrupted alignment and, hence, reflected more severe consequences in relation to identity and well-being. Conversely, the participants in the third narrative expressed higher aspects of self-agency and resilience, such as the ability to endure, cope, reset, and maintain hope, in comparison to the two other composite narratives. However, this clinging on to the “performance narrative” could also be interpreted as an unhealthy part of the “performance narrative,” as living out the “right” way of dealing with injuries or sickness (i.e., being mentally tough, able to tolerate pain, and never ever giving up). The latter interpretation of the narrative aligns with the previously identified “hope and restitution” narrative (Smith and Sparkes, 2005; Kerr and Stirling, 2017) in elite sport settings or the “talent needs trauma” narrative previously identified in TIDS environments (Collins and MacNamara, 2012; Collins et al., 2016; Savage et al., 2017).

The “Just Hanging in There” composite narrative represented a different storyline, revealing more clearly narrative tensions. These junior elite performers faced a lot of trials and many negative consequences, such as identity confusion, meaninglessness, lack of motivation, lack of coping strategies, performance anxiety, and signs of burnout. As the performance identity was so strong and narrow, alongside a lack of agency and inner drive, these performers viewed quitting as not an option and continuously pushed themselves into this seemingly nonoptimal and unhealthy situation. This is, indeed, at odds with the “holistic narrative” that postulates that, to perform well, you need to be well (Miller and Kerr, 2002; Rongen et al., 2014; Wylleman and Rosier, 2016).

### Applied Perspectives

From an applied perspective, it is important to be aware of the pressurized and exposed situations that strained and vulnerable junior elite performers in TIDS might face. Additionally, it seems crucial that coaches and teachers from TIDS become cognizant of the learning conditions they create and the likely consequences (i.e., PE fit and agency). Wylleman and Rosier (2016) discuss the different developmental stages and the instructor’s role in what they call “holistic” talent development. In the transition from adolescence into young adulthood, the performers are required to develop greater independence and agency. Granting autonomy is part of the picture, but more independence and responsibility also require agency (i.e., self-discipline and self-regulation). The ability to see what is needed to make progress may not always be clear for either party, as improvement or development is not a linear process. Based on the present findings, we would argue that wise teachers and coaches see and hear their students and adapt their feedback to their stage in the process and identity development; as such, successful TIDS are very much about individualization and creating a student-centered PE fit.

### Strengths and Limitations

Qualitative data richness and a contextualized narrative approach are strengths in this study, contributing to the “small story” research genre in the case of TIDS. Our study showed the ongoing, interactive negotiation of identity narratives, offering an *in situ* perspective, in contrast to the retrospective perspective. The study has focused on strained junior elite performers to reveal more nuanced knowledge of the ones that do not fit the success stories of the elite performers often told in TIDS context and mainstream media. However, a weakness is that we only rely on interviews with the performers (i.e., no triangulation was done with observations or the leaders’ perspectives). Also, we lack the long-term perspective of these performers’ future positions.

## Concluding Remarks

The present study examined narrative tensions in strained junior elite performers’ experiences of becoming elite performers within the context of TIDS. Indeed, the findings supported results from previous research that emphasized the complex, situated, and dynamic nature of talent development. The findings identified that the “performance narrative” was most dominant in the TIDS, pressurizing the junior elite performers into a predetermined plot of becoming and challenging their identity, agency, and physical and mental health. Our findings demonstrated that the “performance narrative” entailed unhealthy and inhumane aspects, beyond the presence of narrative alignment and PE fit. However, experiences of narrative tensions and a PE misfit seemed to intensify the risks and costs associated with TIDS participation in the strained junior elite performers. Potential alternative narratives and side stories that have the potential to advance the understanding of becoming were also identified, although not fully acknowledged by the TIDS. Hence, in the future, TIDS should adopt a more holistic and flexible approach to constituting and developing elite performers.

## Data Availability Statement

The datasets presented in this article are not publicly available because the participants have been assured full anonymity, and hence, the data will not be passed on to a third party. Requests to access the datasets should be directed to the corresponding author (heidhara@khio.no).

## Ethics Statement

The studies involving human participants were reviewed and approved by Norwegian Center of Research Data (NSD). The patients/participants provided their written informed consent to participate in this study.

## Author Contributions

HMH, FA, and HH conceived the presented idea. HMH, FA, and BS developed the theory. HMH performed the data collections and analysis. FA, BS, and HH peer debriefed and verified the analytical methods and results. HMH, FA, BS, and HH discussed the results and contributed to the final writing of the manuscript. All authors contributed to the article and approved the submitted version.

### Conflict of Interest

The authors declare that the research was conducted in the absence of any commercial or financial relationships that could be construed as a potential conflict of interest.
